# Intravenous Thrombolysis in Posterior versus Anterior Circulation Stroke: Clinical Outcome Differs Only in Patients with Large Vessel Occlusion

**DOI:** 10.3390/biomedicines12020404

**Published:** 2024-02-09

**Authors:** Simona Halúsková, Roman Herzig, Robert Mikulík, Silvie Bělašková, Martin Reiser, Lubomír Jurák, Daniel Václavík, Michal Bar, Lukáš Klečka, Tomáš Řepík, Vladimír Šigut, Aleš Tomek, David Hlinovský, Daniel Šaňák, Oldřich Vyšata, Martin Vališ, on behalf of the Czech SITS Investigators

**Affiliations:** 1 Department of Neurology, Faculty of Medicine in Hradec Králové, Charles University, 500 03 Hradec Králové, Czech Republic; sim.haluskova@gmail.com (S.H.);; 2 Department of Neurology, Faculty of Health Studies, Pardubice University and Pardubice Hospital, 532 10 Pardubice, Czech Republic; 3 Department of Neurology, Comprehensive Stroke Center, University Hospital Hradec Králové, 500 05 Hradec Králové, Czech Republic; 4 Research Institute for Biomedical Science, 500 02 Hradec Králové, Czech Republic; 5 International Clinical Research Center, St. Anne’s University Hospital in Brno, 602 00 Brno, Czech Republic; 6 Department of Neurology, Comprehensive Stroke Center, Hospital České Budějovice, 370 01 České Budějovice, Czech Republic; 7 Neurocenter, Regional Hospital Liberec, 460 63 Liberec, Czech Republic; 8 Department of Clinical Neurosciences, Faculty of Medicine, University of Ostrava, 708 00 Ostrava, Czech Republic; 9 Research and Training Institute Agel, Stroke Center, Department of Neurology, Hospital Ostrava Vítkovice, 703 00 Ostrava, Czech Republic; 10 Department of Neurology, Comprehensive Stroke Center, Faculty of Medicine, University of Ostrava and University Hospital Ostrava, 708 52 Ostrava, Czech Republic; 11 Stroke Center, Department of Neurology, City Hospital Ostrava, 703 00 Ostrava, Czech Republic; 12 Department of Neurology, Comprehensive Stroke Center, University Hospital in Pilsen and Faculty of Medicine in Pilsen, Charles University, 323 00 Pilsen, Czech Republic; 13 Stroke Center, Department of Neurology, Krnov Hospital, 794 01 Krnov, Czech Republic; 14 Department of Neurology, Comprehensive Stroke Center, Charles University 2nd Faculty of Medicine, Motol University Hospital, 150 06 Prague, Czech Republic; 15 Stroke Center, Department of Neurology, Thomayer University Hospital, 140 59 Prague, Czech Republic; 16 Department of Neurology, Comprehensive Stroke Center, Faculty of Medicine and Dentistry, Palacký University and University Hospital Olomouc, 779 00 Olomouc, Czech Republic

**Keywords:** stroke, posterior circulation, anterior circulation, intravenous thrombolysis, intracerebral hemorrhage, clinical outcome

## Abstract

The safety and efficacy of intravenous thrombolysis (IVT) are well established in anterior circulation stroke (ACS) but are much less clear for posterior circulation stroke (PCS). The aim of this study was to evaluate the occurrence of parenchymal hematoma (PH) and 3-month clinical outcomes after IVT in PCS and ACS. In an observational, cohort multicenter study, we analyzed data from ischemic stroke patients treated with IVT prospectively collected in the SITS (Safe Implementation of Treatments in Stroke) registry in the Czech Republic between 2004 and 2018. Out of 10,211 patients, 1166 (11.4%) had PCS, and 9045 (88.6%) ACS. PH was less frequent in PCS versus ACS patients: 3.6 vs. 5.9%, odds ratio (OR) = 0.594 in the whole set, 4.4 vs. 7.8%, OR = 0.543 in those with large vessel occlusion (LVO), and 2.2 vs. 4.7%, OR = 0.463 in those without LVO. At 3 months, PCS patients compared with ACS patients achieved more frequently excellent clinical outcomes (modified Rankin scale [mRS] 0–1: 55.5 vs. 47.6%, OR = 1.371 in the whole set and 49.2 vs. 37.6%, OR = 1.307 in those with LVO), good clinical outcomes (mRS 0–2: 69.9 vs. 62.8%, OR = 1.377 in the whole set and 64.5 vs. 50.5%, OR = 1.279 in those with LVO), and had lower mortality (12.4 vs. 16.6%, OR = 0.716 in the whole set and 18.4 vs. 25.5%, OR = 0.723 in those with LVO) (*p* < 0.05 in all cases). In PCS versus ACS patients, an extensive analysis showed a lower risk of PH both in patients with and without LVO, more frequent excellent and good clinical outcomes, and lower mortality 3 months after IVT in patients with LVO.

## 1. Introduction

Intravenous thrombolysis (IVT) with the administration of recombinant tissue plasminogen activator (rtPA) is an evidence-based reperfusion therapy and remains the standard care protocol for patients with acute ischemic stroke (AIS) [1,2]. Data from randomized clinical trials have supported the beneficial effect of IVT on outcome measures when initiated within 4.5 h of clearly defined symptom onset [3,4], or even up to 9 h in carefully selected patients based on advanced imaging techniques [5]. Patients with both anterior (ACS) and posterior circulation stroke (PCS) could have been enrolled in these studies, but whether the efficacy and safety differ between ACS and PCS has not been reported, and high-level evidence for the evaluation of post-IVT outcomes in PCS alone is limited. PCS represents 12–19% of all IVT-treated strokes [6,7,8,9] and it is even more frequent in the non-IVT population (20–26%) [6]. Although data from real-world registries and observational studies have indicated that patients with PCS might benefit from IVT, analyzed numbers have been small (30 to 124) and the results controversial [6,7,8,10,11,12,13,14,15]. Therefore, it remains unclear whether outcomes in AIS patients treated with IVT can be influenced based on the affected vascular territory. We hypothesized that patients with PCS may have a different tendency to develop intracerebral parenchymal hematoma (PH) after IVT than patients with ACS due to the developmental difference in the vascular supply of the anterior and posterior cerebral circulation [16,17].

The present study aimed to assess overall differences between PCS and ACS patients after IVT, as well as in particular subgroups of patients with and without large vessel occlusion (LVO), in terms of the occurrence of PH and 3-month clinical outcomes. 

## 2. Materials and Methods

### 2.1. Patients

In this observational, nationwide, multicenter study, we analyzed data for all adult patients who underwent IVT for AIS with a standard dose of 0.9 mg/kg of rtPA (Actilyse^®^; Boehringer Ingelheim, Ingelheim am Rhein, Germany) within 4.5 h of symptom onset following the national guidelines [18,19,20], prospectively collected in the SITS (Safe Implementation of Treatments in Stroke) registry in the Czech Republic between January 2004 and December 2018. According to the uniform protocol, the clinical and demographic data of patients treated with IVT in the Czech Republic are stored in the SITS registry. The SITS registry is a prospective, multicenter international data collection platform designed to confirm the safety and efficacy of IVT in clinical practice [21]. The registry was used in the Czech Republic between 2004 and 2018.

Patients were considered eligible for analysis when data on a particular stroke either in the posterior or anterior circulation (patients with unclear stroke territory and acute infarctions in both circulation territories were not included), information on the presence of intracerebral hemorrhage (ICH) on a non-enhanced brain computed tomography (CT) scan performed 22–36 h after IVT, as well as information on the 3-month clinical outcomes were entered in the registry. Patients undergoing mechanical thrombectomy were not included to ensure the homogeneity of the set. Stroke centers that reported fewer than 30 patients were excluded from the statistical point of view because only 30 reports or more per center are enough to calculate the intraclass correlation coefficient (ICC) and consider how big the hospital’s effect is. No additional exclusion criteria were imposed. Enrolled patients were divided into the PCS or ACS groups. PCS was defined as symptomatic ischemia occurring in the vascular territory of the vertebral (VA), basilar (BA), or posterior cerebral arteries (PCA) and their branches. ACS was classified as symptomatic ischemia involving the territory of the internal carotid (ICA), middle cerebral (MCA), or anterior cerebral arteries (ACA) and their branches.

This observational study’s reporting follows the Strengthening the Reporting of Observational Studies in Epidemiology (STROBE) guidelines.

### 2.2. Baseline Data

A large range of parameters was gathered and prospectively recorded in the SITS registry in a prespecified manner and then analyzed retrospectively. The following variables were used for the analysis: (A) demographic data on patient sex and age; (B) history (any time before the index AIS) of arterial hypertension (including patients on antihypertensive therapy who were normotensive at the time of the index AIS), congestive heart failure, atrial fibrillation, diabetes mellitus, hyperlipidemia, obesity (defined as body mass index ≥30 kg/m^2^), current smoking (at the time of index AIS onset), previous (any time before the index AIS) stroke or transient ischemic attack (TIA), and information on pre-admission pharmacotherapy including the use of anticoagulation, antiplatelet, and statin therapy; (C) baseline neurological deficit assessed using the National Institutes of Health Stroke Scale (NIHSS) score [22], involved vascular territory (anterior/posterior), detection of LVO on the initial CT angiography (LVO was characterized as the occlusion of the extracranial carotid artery, intracranial ICA, A1 and A2 segments of the ACA, M1 and M2 segments of the MCA, extracranial VA, intracranial VA, BA, P1 and P2 segments of the PCA, concurrent occlusions of multiple of the aforementioned arteries, and also tandem pathology defined as a combination of ICA and M1/M2 segment occlusion), serum glucose and cholesterol levels, systolic and diastolic blood pressure values, need for intravenous antihypertensive therapy before/during IVT administration, and onset-to-needle (ONT) and door-to-needle (DNT) times evaluated on the basis of available data on the time of symptom onset, time of arrival to the hospital and time of IVT bolus dose administration; and (D) post-stroke prophylaxis of *deep venous thrombosis* with *low-molecular-weight heparin (LMWH)*, and *treatment* with antiplatelet drugs and oral anticoagulants.

### 2.3. Outcome Parameters

The primary outcome measure was an intracerebral PH, defined as any non-petechial intraparenchymal bleeding as evidenced by the routine follow-up brain CT scan 22–36 h after IVT [8] (performed in all patients and evaluated by a certified radiologist and certified neurologist), regardless of clinical deterioration. To ensure the homogeneity of the sample, patients with hemorrhagic infarction type I and type II (i.e., petechial hemorrhages) [23] were not included in the analysis. Unfortunately, data were unavailable for some important clinical parameters, and due to a lack of available data about 24-h (n = 829) and 7-day (n = 585) NIHSS scores we were not able to assess the rates of symptomatic ICH (SICH) after IVT according to the SITS-MOST (Safe Implementation of Thrombolysis in Stroke Monitoring Study) [24], ECASS II (Second European Co-operative Stroke Study) [23], and NINDS (Neurological Disorders and Stroke Definition) [25] criteria. Moreover, we considered the evaluation of PH to be more accurate and independent of the assessment of the patient’s clinical state. The secondary endpoint was 3-month clinical outcome assessed by a certified neurologist using the modified Rankin Scale (mRS) with excellent clinical outcome defined as 0–1 points, good clinical outcome (functional independence) as 0–2 points, and mortality defined as mRS 6 [26].

### 2.4. Statistical Analysis

**Figure 1:**
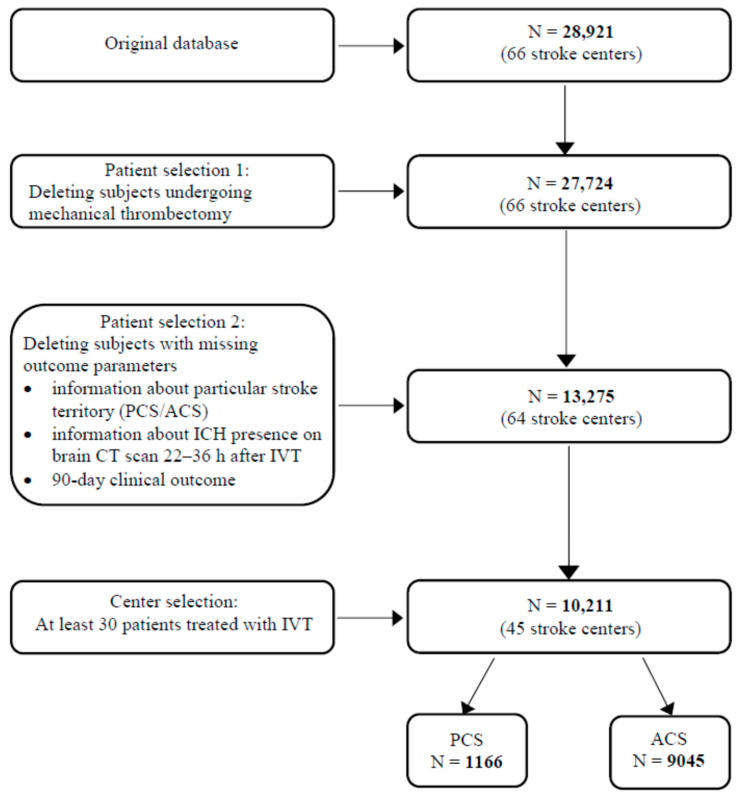
Study flowchart. ACS—anterior circulation stroke; CT—computed tomography; ICH—intracerebral hemorrhage; IVT—intravenous thrombolysis; N—number of subjects; PCS—posterior circulation stroke.

Due to a large number of missing values, a multi-step data cleaning process was performed, involving the removal of subjects for whom no data were available regarding the target variables, i.e., affected vascular territory (anterior or posterior), IVT-related ICH, and 3-month mRS. Finally, only centers with at least 30 observations were considered for the analysis (Figure 1).

In the case of categorical variables, data are presented as a number of observations and percentages and a *p* value for hypothesis testing. Whether the differences between the PCS and ACS groups (including patients with and without LVO) were statistically significant was tested using Fisher’s exact test. If the variable was continuous, we presented the data as mean and standard deviation. For testing the differences between the PCS and ACS groups, the two-sample t-test was used. NIHSS value is presented as median and interquartile range, and the Mann–Whitney test (non-parametrical) was applied to compare the two groups. The reported *p* values are two-tailed, and a 5% significance level was chosen. N-used is the number of observations used for the analysis.

Implementation of Logistic Regression for Propensity Score Matching

In our study, we employed logistic regression to estimate the propensity scores for each patient, which is crucial for reducing selection bias in observational studies. This methodological choice was grounded in its ability to model the probability of stroke localization (ACS vs. PCS) based on observed characteristics.

Logistic Regression Model Specification

We defined stroke localization assignment as the dependent variable in our logistic regression model. The independent variables included a comprehensive range of observed characteristics, such as demographic data (age, sex), clinical parameters (stroke severity, ONT), risk factors (history of arterial hypertension, diabetes, etc.), and pharmacotherapy used before the stroke. These variables were selected based on their potential influence on the likelihood of stroke localization.

Estimation of Propensity Scores

The logistic regression model provided us with propensity scores—the probability of stroke localization in each patient given their observed characteristics. These scores were essential for matching patients in the ACS group with those in the PCS group who had similar propensity scores. This matching process aimed to create a balanced comparison.

Model Diagnostics and Validation

To ensure the robustness of our logistic regression model, we conducted several diagnostic tests. These included assessments of model fit, multicollinearity among independent variables, and the overall predictive power of the model. The validity of the model was further substantiated by checking the balance of covariates across the ACS and PCS groups post-matching.

Significance of Logistic Regression in Our Analysis

The use of logistic regression for propensity score estimation allowed us to address potential confounding factors and selection biases inherent in observational studies. By effectively balancing the ACS and PCS groups regarding their baseline characteristics, we enhanced the credibility of our findings, providing a more reliable estimate of stroke localization.

First of all, ICC was calculated from a model with fixed effects for the main outcome PH and also mRS 0–1. Initially, all predictive variables were considered possible predictors. Logistic regression model implementation included two steps. Firstly, we created a predictive model based on a stepwise regression forward and backward. Results from those models were then collected and fixed effects were put into two separate models—one with and one without the random effect of the center. Because there was only a small inner correlation, the results from the logistic model were used with a 95% confidence interval based on Wald test statistics including correction for multiple comparisons. The odds ratio has been adjusted to account for predictor variables with significantly different representations in Table 1. Some confounders were removed according to “The 10% rule”. Analysis was conducted in the statistical software R 1.6 (R Foundation for Statistical Computing, Vienna, Austria) and Python 3 (Python Software Foundation, Waltham, MA, USA).

## 3. Results

The original database contained clinical records for 28,921 subjects from 66 Czech stroke centers. In the course of the data cleaning process, our final set consisted of 10,211 eligible adult patients. Of these, 1166 (11.4%) had PCS and 9045 (88.6%) had ACS. Baseline characteristics of the study population including demographics, vascular risk factors, clinical parameters, and pre-stroke and post-stroke pharmacotherapy are presented in Table 1. Patients with PCS were significantly younger, less frequently female, and had a more favorable cardiovascular risk factor profile than ACS patients. While patients in the PCS group used antiplatelet drugs and statins before AIS less often compared to those in the ACS group, post-stroke medication, namely antiplatelets and prophylactic LMWH, was more frequent in PCS patients. LVO was detected significantly more frequently in PCS patients. PCS patients presented with a milder neurological deficit on admission, had higher baseline blood glucose levels, and were more likely to experience longer ONT and DNT.

**Table 1:** Baseline variables.

Observed Parameter	n-Used	PCS Group n = 1166 (11.4%)	ACS Group n = 9045 (88.6%)	*p*
Demographics				
Women; n (%)	10,211	459 (39.4)	4151 (45.9)	**<0.001**
Age; years, mean (SD)	10,199	67.1 (13.3)	70.7 (12.4)	**<0.001**
Occurrence of vascular risk factors				
Arterial hypertension; n (%)	10,043	821 (71.5)	6794 (76.4)	**0.0003**
Congestive heart failure; n (%)	10,003	64 (5.6)	805 (9.1)	**<0.0001**
Atrial fibrillation; n (%)	10,039	197 (17.2)	2000 (22.5)	**<0.0001**
Diabetes mellitus; n (%)	10,156	298 (25.6)	2623 (29.2)	**0.012**
Hyperlipidemia; n (%)	9925	398 (34.8)	3108 (35.4)	0.718
Obesity; n (%)	6197	236 (29.3)	1476 (27.4)	0.254
Current smoking; n (%)	9393	213 (19.3)	1500 (18.1)	0.319
Previous stroke or TIA; n (%)	10,118	207 (17.9)	1643 (18.3)	0.716
Pre-stroke pharmacotherapy				
Anticoagulation; n (%)	9502	45 (4.1)	401 (4.8)	0.326
Antiplatelets; n (%)	10,140	385 (33.1)	3518 (39.2)	**<0.001**
Statins; n (%)	9434	324 (27.9)	2574 (31.1)	**0.029**
Clinical parameters				
Baseline NIHSS; median (IQR)	9918	6 (4–9)	8 (5–14)	**<0.0001**
Large vessel occlusion; n (%)	6164	423 (52.9)	2451 (45.7)	**0.0002**
Glucose; mmol/L, mean (SD)	9642	8.0 (2.9)	7.7 (2.8)	**0.0001**
Cholesterol; mmol/L, mean (SD)	7016	4.9 (1.2)	4.8 (1.2)	0.172
SBP; mmHg, mean (SD)	9941	158.0 (26.3)	159.4 (24.8)	0.103
DBP; mmHg, mean (SD)	9941	85.6 (14.5)	86.1 (14.2)	0.291
i.v. antihypertensive therapy before/during IVT; n (%)	9584	191 (17.4)	1461 (17.2)	0.899
ONT; min, mean (SD)	9779	160.8 (76.3)	144.7 (60.9)	**<0.0001**
DNT; min, mean (SD)	10,013	54.6 (37.2)	51.2 (32.3)	**0.003**
Post-stroke pharmacotherapy				
LMWH; n (%)	9113	644 (57.8)	4267 (53.3)	**0.005**
Anticoagulation; n (%)	8872	90 (8.1)	577 (7.4)	0.430
Antiplatelets; n (%)	9477	758 (65.2)	4989 (60.0)	**0.0008**

Statistically significant differences are highlighted in bold. ACS—anterior circulation stroke; DBP—diastolic blood pressure; DNT—door-to-needle time; IQR—interquartile range; i.v.—intravenous; IVT—intravenous thrombolysis; LMWH—*low-molecular-weight heparin*; n—number; NIHSS—National Institutes of Health Stroke Scale; ONT—onset-to-needle time; PCS—posterior circulation stroke; SBP—systolic blood pressure; SD—standard deviation; TIA—transient ischemic attack.

**Figure 2:**
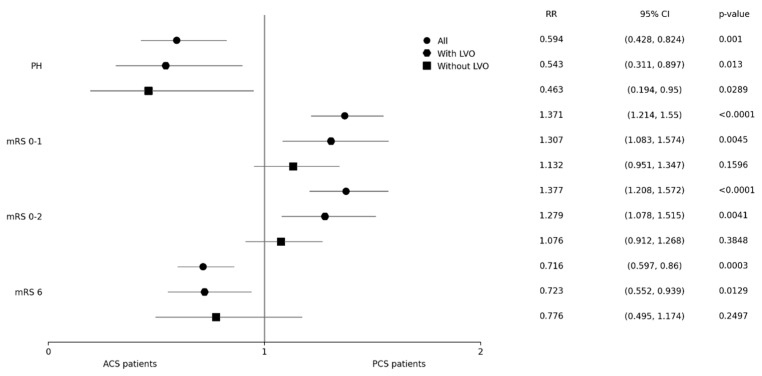
Primary and secondary outcomes. ACS–anterior circulation stroke; CI–confidence interval; LVO–large vessel occlusion; mRS–modified Rankin scale; OR–odds ratio; PH–parenchymal hematoma; PCS–posterior circulation stroke.

Primary and secondary outcome measures regarding PH and mRS among PCS and ACS patients (both with and without LVO) are shown in Figure 2 and Table 2. Concerning the occurrence of ICH, only patients with PH were included in the analysis (308 patients with hemorrhagic infarction type I and 208 with hemorrhagic infarction type II were not included). PH was noted significantly less often in the PCS group, including subgroups of patients both with and without LVO. In PCS patients, the odds for PH were approximately 1.6 times lower as compared to patients with ACS. PCS patients had significantly better 3-month clinical outcomes and also lower 3-month mortality. In the analysis of subgroups, this only applied to patients with LVO and not to patients without LVO.

Finally, we tried to determine independent predictors of PH occurrence, excellent and good 3-month clinical outcomes, and mortality. The results of binary logistic regression analysis are depicted in Table 3. No association between stroke territory and outcome parameters of interest was found.

## 4. Discussion

According to the authors’ knowledge, this is the first study assessing the safety of IVT in PCS and ACS separately in the subgroups of patients both with and without LVO and only the second one assessing the outcomes in these patients [27].

As a consequence of the emphasis of large thrombolysis trials on ACS [23,25,28], our knowledge concerning the potential benefits and risks of IVT in PCS patients, in general, is limited. Such an imbalance is mainly due to the small proportion of PCS, the wide variety of anatomical structures supplied by the vertebrobasilar system that is associated with a huge diversity of clinical syndromes, and, last but not least, as repeatedly discussed, the inadequate representation of PCS symptoms by the NIHSS resulting in overall lower NIHSS scores in patients with PCS compared to ACS [7,13,29,30,31,32,33,34,35]. PCS, therefore, remains out of the spotlight for stroke neurologists. Until now, several studies have investigated the safety and clinical outcomes after IVT in PCS. Most of them had small sample sizes, assessed intracranial hemorrhage using different classifications, or were restricted to patients with basilar artery occlusion [6,7,9,12,15,36,37].

In line with some previous observations, our PCS patients were younger and more often males [6,8,27,38], had less severe baseline NIHSS scores [7,8,10,12,13,14,38,39], lower occurrence of atrial fibrillation [6,8,9,10,15,27,38,39], higher blood glucose levels [6,8,15] and slower ONT and DNT [8,13] in comparison to ACS patients. The reported occurrence of other conventional stroke risk factors, clinical parameters, and prophylactic treatment recorded at admission or discharge in both groups has varied widely across studies with diverse degrees of statistical significance.

ICH is considered to be the most serious complication following the administration of IVT in patients with AIS. Unlike several studies that assessed SICH per the SITS-MOST, ECASS II, or NINDS criteria [7,8,9,12,13,15,40], we noted any non-petechial intraparenchymal bleeding as the main safety endpoint. Concerning the above-mentioned definitions counting on the change of NIHSS value, they are to some extent dependent on the subjective evaluation of the physician, and the reason for apparent clinical worsening could also be caused by non-neurological conditions or in some specific situations it may not be validly estimated at all (e.g., due to artificial sedation, coma, or need for endotracheal intubation). To compare ICH risks, it is more relevant to include all radiologically proven post-IVT hemorrhagic complications. In the present study, the rate of PH was lower in PCS patients, which is consistent with data reported by Sung et al. [9], Dorňák et al. [6], Tong et al. [15] (although PH characterization in these papers was based on the ECASS radiological classification [23,28] of the hemorrhagic transformation of an infarct and did not involve remote PH), and also with work by Keselman et al. [8] Unlike the latter already mentioned study [8] which included only patients with arterial occlusion proven by CT angiography or magnetic resonance angiography, we demonstrated that PH was significantly less frequent in the PCS group, including subgroups of patients both with and without LVO. Last but not least, PCS was associated with a decreased risk for all-type ICH according to a meta-analysis performed by Lee et al. [41] The reason for the documented overall lower occurrence of ICH in PCS has been widely debated in the literature and remains rather hypothetical. However, a few theories have been proposed in recent years to help explain this phenomenon. First, collateral supply in the posterior circulatory system might be better than that in the anterior circulation [17]; it has been demonstrated that patients with good collaterals were less prone to hemorrhagic complications after acute recanalization therapy—both IVT and endovascular treatment [42,43]. Second, better collateral circulation likely results in a slower evolution of irreversible ischemia and smaller infarct volumes in PCS which may be partly associated with lower bleeding rates as well [41,44,45]. Third, some previous studies indicated different histopathological changes in the brain tissue after stroke onset among the two territories with probable delayed blood–brain barrier disruption in PCS, which may also contribute to decreased risk of subsequent hemorrhage [16,46].

Nevertheless, data regarding clinical outcomes differ. Earlier works have reported conflicting results on whether the functional outcome of PCS is comparable to ACS. Historically, patients with PCS have been thought to have a poorer outcome with high morbidity and mortality [47,48], but latter studies have shown the opposite to be true. Most of the studies have found that the 3-month outcomes after IVT treatment alone in patients with PCS were better or very much alike to ACS patients, including the significantly more frequent achievement of both excellent (55.7 vs. 41.6% [15] and 45.2 vs. 37.5% [8]) and good (63.9 vs. 53.0% [15], 61.3 vs. 49.4% [8] and 93.8 vs. 70.2% [14], respectively) outcomes. These results run parallel with our findings. On the other hand, no significant differences were noted regarding excellent recovery or functional independence at 3 months between these particular patient groups in other works [7,10,13,37,40], and, inconsistent with our results, 3-month mortality also did not significantly differ between PCS and ACS patients [8,10,12,15,37]. On the contrary, Kim et al. reported significantly higher odds of disabled outcome (defined as mRS 2–6) in minor PCS with an NIHSS value of ≤4 at baseline as compared to minor ACS (adjusted odds ratio [OR] 1.23) [27]. This work is, to the best of our knowledge, the only study that included pairwise comparisons of 3-month outcomes in ACS and PCS patient groups according to the presence or absence of major large vessel disease, defined as symptomatic stenosis of >50% or occlusion of the artery (MCA, ICA, VA, and BA) supplying the ischemic area. Also, exclusively patients with low NIHSS scores (≤4 at baseline) were enrolled. The authors demonstrated that patients with minor PCS with large vessel disease were more likely to be disabled (mRS 2–6; OR 1.58) and dependent (mRS 3–6; OR 1.36) at 3 months compared with patients with minor ACS with large vessel disease [27]. Our PCS patients with LVO experienced significantly better 3-month clinical outcomes and also lower 3-month mortality than ACS patients with LVO. However, these differences were not found in our PCS and ACS subgroups without LVO.

Despite the fact we did not evaluate stroke etiology, evidence from other studies exists, pointing out that cardioembolism is more frequent in ACS [11,27,40,49]. On the other hand, some data suggest that large-artery atherosclerosis as a causative mechanism of ischemic *stroke* is more common in PCS [27]. We assume this is analogous for our PCS group, which is indirectly indicated by the significantly higher use of antiplatelets after stroke in PCS as compared to ACS, whilst the ratio was reversed before the event. Subsequent secondary prevention with antiplatelets might also positively improve outcomes by reducing the likelihood of recurrent stroke.

The knowledge of predictors for ICH and clinical outcomes following IVT can be extremely useful, especially in certain specific situations and borderline cases requiring therapeutic decisions; hence, it is an issue often discussed in the literature [6,10,12,37,50,51,52]. Dorňák et al. identified ACS as a significant predictor of a large hemorrhage (PH1 + PH2 according to the ECASS radiological classification [6,23,28]). Surprisingly, although ACS was considerably more risky regarding the occurrence of PH, 3-month mortality rates, and lower achievement of mRS 0–1 and 0–2 in the present study, the effect of particular circulation was not identified as an independent risk factor for the observed parameters in the performed logistic regression analysis. In addition, Sarikaya et al. found no association between either stroke territory or mortality nor between circulation and excellent 3-month clinical outcome [12]. Similar results were reported in the work by Jalali et al., in which the arterial territory was not an outcome predictor in AIS—there was no significant association between ACS/PCS and mortality, excellent clinical outcome, functional independence, and ICH [53].

The strength of our study is the large sample size with a sufficient number of both PCS and PH patients used for the analysis. Insufficient numbers were often an issue that complicated the application of binary logistic regression models in smaller series with modest sample sizes. Another strength is multicenter data collection over more than 10 years, a relatively high number of variables analyzed including the presence of LVO, and the nationwide character of the population. Even though some extensive meta-analyses dealing with similar topics have been published [8,41], to the best of our knowledge this is the largest registry-based study comparing the safety and clinical outcomes after IVT between PCS and ACS patients, with the sample size clearly outnumbering the majority of previous works.

The limitations of the present study primarily result from the fact that it was based on data contributed to the registry from multiple centers voluntarily. Therefore, there is no guarantee that all the data on all AISs were inserted into the register and it is not possible to determine how many patients were not reported to the database and because of what reasons or why there are missing values for particular variables. Unfortunately, data were also missing in the case of some important clinical parameters; for this reason, we were not able to assess the rates of SICH per the SITS-MOST, ECASS II, and NINDS definitions. Still, as we analyzed data from 2004 to 2018, there was a clear trend toward a lower number of missing values over the years. It is undoubtedly true that registers like the SITS provide us with valuable data and are essential for the judicious evaluation of AIS care quality. Secondly, data collection methods among databases may be the source of selection bias, which we cannot rule out. The retrospective, observational, nonrandomized nature of this study’s design is another limitation as well as the absence of data on ethnicity, although the vast majority of the inhabitants of the Czech Republic are Caucasians.

## 5. Conclusions

In summary, data on thrombolyzed patients collected in the SITS registry showed that PCS was associated with a significantly lower risk of PH occurrence and, in patients with LVO, also with higher rates of excellent and good 3-month clinical outcomes and decreased mortality at 3 months as compared with ACS. Thus, IVT is likely to be safer in PCS than in ACS patients and 3-month clinical outcomes of IVT in PCS appear to be at least comparable to those of ACS patients.

## Figures and Tables

**Table 2:** Primary and secondary outcomes.

Observed Parameter n (%)	n-Used	PCS Group All 1166 (11.4)	ACS Group All 9045 (88.6)	*p*	n-Used	PCS Group with LVO 423 (36.3)	ACS Group with LVO 2451 (27.1)	*p* <0.0001	PCS Group without LVO 377 (32.3)	ACS Group without LVO 2913 (32.2)	*p* 0.9498
PH	9695	40 (3.6)	505 (5.9)	**0.001**	5854	18 (4.4)	178 (7.8)	**0.013**	8 (2.2)	131 (4.7)	**0.0289**
mRS 0–1	10,211	647 (55.5)	4307 (47.6)	**<0.0001**	6164	208 (49.2)	922 (37.6)	**0.0045**	251 (66.6)	1713 (58.8)	0.1596
mRS 0–2	10,211	815 (69.9)	5676 (62.8)	**<0.0001**	6164	273 (64.5)	1237 (50.5)	**0.0041**	299 (79.3)	2148 (73.7)	0.3848
mRS 6	10,211	145 (12.4)	1497 (16.6)	**0.0003**	6164	78 (18.4)	625 (25.5)	**0.0129**	27 (7.2)	269 (9.2)	0.2497

Statistically significant differences are highlighted in bold. ACS—anterior circulation stroke; LVO—large vessel occlusion; mRS—modified Rankin scale; n—number; PCS—posterior circulation stroke; PH—parenchymal hematoma.

**Table 3:** Adjusted results of binary logistic regression analysis—independent predictors of intracerebral parenchymal hematoma and 90-day clinical outcomes.

Observed Parameter	OR	95% CI	*p* Value
Predictors of the PH			
Age	1.020	1.005–1.035	**0.009**
NIHSS	1.043	1.017–1.069	**0.001**
i.v. antihypertensive therapy before/during IVT	1.729	1.216–2.459	**0.002**
Predictors of mRS 0–1			
Age	0.968	0.962–0.974	**<0.0001**
Previous stroke or TIA	0.723	0.612–0.855	**<0.0001**
NIHSS	0.861	0.849–0.873	**<0.0001**
Large vessel occlusion	0.660	0.578–0.754	**<0.0001**
Glucose level	0.937	0.915–0.959	**<0.0001**
i.v. antihypertensive therapy before/during IVT	0.581	0.482–0.700	**<0.0001**
ONT	0.998	0.997–1.000	**0.005**
Post-stroke prophylaxis of *deep venous thrombosis* with *LMWH*	0.834	0.732–0.950	**0.006**
Predictors of mRS 0–2			
Age	0.950	0.943–0.957	**<0.0001**
Atrial fibrillation	0.770	0.640–0.926	**0.006**
NIHSS	0.858	0.846–0.870	**<0.0001**
Large vessel occlusion	0.630	0.543–0.730	**<0.0001**
Glucose level	0.914	0.891–0.937	**<0.0001**
i.v. antihypertensive therapy before/during IVT	0.558	0.465–0.671	**<0.0001**
ONT	0.998	0.996–0.999	**<0.0001**
Predictors of mRS 6			
Female sex	0.703	0.558–0.885	**0.003**
Age	1.080	1.066–1.094	**<0.0001**
Atrial fibrillation	1.399	1.010–1.781	**<0.006**
NIHSS	1.146	1.125–1.167	**<0.0001**
Large vessel occlusion	2.057	1.624–2.605	**<0.0001**
Glucose level	1.115	1.077–1.156	**<0.0001**
Cholesterol level	0.852	0.770–0.942	**0.002**
i.v. antihypertensive therapy before/during IVT	2.121	1.648–2.729	**<0.0001**
ONT	1.004	1.002–1.005	**<0.0001**

Statistically significant differences are highlighted in bold.CI—confidence interval; i.v.—intravenous; IVT—intravenous thrombolysis; LMWH—*low-molecular-weight heparin*; mRS—modified Rankin scale; NIHSS—National Institutes of Health Stroke Scale; ONT—onset-to-needle time; OR—odds ratio; PH—parenchymal hematoma; TIA—transient ischemic attack.

## Data Availability

The datasets generated during and/or analyzed during the current study are available from the corresponding author upon reasonable request.
